# Impact of early surfactant and inhaled nitric oxide therapies on outcomes in term/late preterm neonates with moderate hypoxic respiratory failure

**DOI:** 10.1038/jp.2013.83

**Published:** 2013-07-18

**Authors:** G G Konduri, G M Sokol, K P Van Meurs, J Singer, N Ambalavanan, T Lee, A Solimano

**Affiliations:** 1 Division of Neonatology, Department of Pediatrics, Medical College of Wisconsin, Milwaukee, WI, USA; 2 Department of Pediatrics, Indiana University School of Medicine, Indianapolis, IN, USA; 3 Department of Pediatrics, Stanford University, Palo Alto, CA, USA; 4 Center for Health Evaluation and Outcome Sciences, St Paul's Hospital, Vancouver, BC, Canada; 5 Department of Pediatrics, University of Alabama at Birmingham, Birmingham, AL, USA; 6 Department of Pediatrics, University of British Columbia, Vancouver, BC, Canada

**Keywords:** newborn, lung disease, persistent pulmonary hypertension of the newborn, PPHN, ECMO

## Abstract

**Objective:**

We conducted a *post-hoc* analysis of early inhaled nitric oxide (iNO)-randomized controlled trial data to identify associations pertinent to the management of moderate hypoxic respiratory failure in term/late preterm infants.

**Study design:**

Univariate and multivariate logistic regression analyses were used to determine risk factors for the progression of respiratory failure and extracorporeal membrane oxygenation (ECMO)/death.

**Result:**

Among the 299 enrolled infants, oxygenation index (OI) <20 at enrollment (odds ratio 0.52, confidence interval (CI) 0.27 to 0.97) and surfactant use before randomization (odds ratio 0.47, CI 0.24 to 0.91) were associated with decreased ECMO/death rates. Early surfactant use for respiratory distress syndrome, perinatal aspiration syndrome and pneumonia/sepsis was associated with lower risk of ECMO/death (*P*<0.001). Early iNO (OI 15 to 25) decreased the progression of respiratory failure to OI >30 (*P*=0.002) and to composite outcome of OI >30 or ECMO/death (*P*=0.02).

**Conclusion:**

This *post-hoc* analysis suggests that early use of surfactant and iNO in moderate respiratory failure is associated with improved outcomes.

## Introduction

Hypoxic respiratory failure (HRF) occurs due to a variety of lung diseases in full-term and late preterm newborn infants.^1, 2^ Perinatal aspiration syndrome, respiratory distress syndrome (RDS), pneumonia/sepsis and primary pulmonary hypertension (no parenchymal lung disease) are the most common lung diseases associated with HRF in term and late preterm neonates.^1, 2, 3^ The course of HRF is often complicated by persistent pulmonary hypertension of the newborn (PPHN) at this gestational age.^1, 2, 3^ Inhaled nitric oxide (iNO) therapy decreases the risk of extracorporeal membrane oxygenation (ECMO)/mortality in neonates with HRF and PPHN at ⩾34 weeks of gestation;^4, 5, 6, 7^ however, the optimum severity of illness to initiate this therapy relative to other supportive measures remains unclear. Despite a decrease in mortality and ECMO use over the last 10 years,^2, 8^ the risk of these complications remains higher for late preterm infants compared with term infants with HRF.^9^ In addition, neonates with HRF can be very labile and early identification of the risk factors for ECMO/death would facilitate their timely transfer to ECMO referral centers.

We previously reported the results of a randomized controlled trial (RCT) of early iNO or placebo (control) in full-term or late preterm gestational age (⩾34 weeks) neonates with a moderate degree of HRF, defined as an oxygenation index (OI) of ⩾15 and <25.^10^ The control group infants were eligible to receive standard iNO therapy if their HRF progressed to an OI of ⩾25. The trial did not show a difference in the primary outcome of ECMO/death between the early iNO and control groups. However, the mean OI at the initiation of iNO for neonates randomized to the early iNO group was 19.6±3, providing limited differentiation in the severity of respiratory failure at iNO initiation from the control group, which was eligible to receive iNO at an OI of ⩾25, and probably affecting the study conclusion. This study remains the largest prospectively collected data set of late preterm and term neonates with moderate HRF from which new information on the management strategies of moderate HRF can be obtained by secondary data analysis. Previous studies suggested that the use of surfactant and high-frequency ventilation (HFV) for HRF in term neonates could alter the progression of respiratory failure and need for ECMO.^11, 12, 13, 14, 15, 16^ Although the early iNO RCT did not require the use of these therapies, it encouraged their use before randomization into the trial.

We hypothesized that the use of surfactant, HFV and early iNO therapy, either alone or in combination, would decrease the risks of ECMO/death in neonates with moderate HRF. To test this hypothesis, we performed a *post hoc*, subgroup analysis of prospectively collected data from the early iNO RCT to identify factors associated with ECMO/death and progression of HRF.

## Methods

The early iNO RCT was approved by Institutional Review Boards at all the participating centers (listed in the appendix) and was registered at clinical trials.gov (NCT00005773). The trial was monitored by an independent data safety monitoring board and its short- and long-term outcomes were previously reported.^10, 17^ The RCT enrolled 299 neonates into two treatment arms between July 1998 and March 2001. Neonates were eligible if they were delivered at ⩾34 weeks gestation, required ventilator support for HRF with an FiO_2_ ⩾0.80 and had an OI ⩾15 and <25 on any two arterial blood gases done at least 15 min, but no more than 12 h apart. Exclusion criteria were as follows: postnatal age >14 days, congenital heart disease other than patent foramen ovale or patent ductus arteriosus, congenital diaphragmatic hernia, life-threatening congenital malformations or prior exposure to iNO therapy. Study gas assignment was double masked; iNO was started at 5 ppm and was increased to 20 ppm if the increase in PaO_2_ was ⩽20 torr. If the baby had <10 torr increase in PaO_2_ at 20 ppm, the dose was returned to 5 ppm. All study subjects were continued on the assigned dose of study gas until they were weaned off or were exited to standard iNO therapy at an OI of ⩾25 or they met the primary study outcome of ECMO and/or death before discharge from the hospital. The secondary analysis of data from this RCT is described below.

### Statistical analysis

We used univariate analysis to identify variables associated with the outcome of ECMO/death and multivariate logistic regression analysis to determine the independent associations of these variables with ECMO/death. Variables were selected *a priori* for possible secondary data analysis at the time of study initiation and included: gestational age, OI at enrollment, PaO_2_ response to study gas, primary diagnosis associated with HRF and treatment with HFV or surfactant before randomization. We used receiver operating characteristic curve analysis to determine the optimal cutoff value for the severity of respiratory failure, defined by OI at the time of enrollment in the study, and to predict ECMO/death. In addition, we evaluated the influence of surfactant on individual lung diseases associated with HRF and the interaction between surfactant and exposure to iNO therapy. We investigated the effect of iNO treatment given at OI of 15 to 25 on the rate of progression of HRF to OI ⩾30 and to a composite outcome of progression to OI ⩾30 and/or ECMO/death. Finally, we assessed the influence of these treatments on the duration of mechanical ventilation, oxygen therapy and time to discharge home by univariate Kaplan–Meier survival curve analysis and a multivariate Cox proportional hazards model. For time to discharge home, infants who died before discharge were treated as never discharged from the hospital.

## Results

### Baseline variables

As previously reported, there were no differences in the baseline variables, including pre-randomization therapies or distribution of lung diseases associated with HRF between the 150 early iNO (iNO at OI 15 to 25) and 149 control group (iNO if OI progression to >25) infants.^10^ Surfactant was given to 64% (192 of 299) of study infants (63% of the early iNO group and 65% of the control group) and HFV was used in 43% of study infants (41% of the early iNO group and 45% of the control group) before randomization. Of the 192 infants treated with surfactant, 44 infants received additional doses after randomization (22 infants in each group). The mean OI at the time of randomization was 19.4±2.8 and was similar between the early iNO group (19.6±3) and control (19.2±2.6) groups. Median age at randomization (for the early iNO group: 28.4 h, with an interquartile range (IQR) of 14 to 46 h; for the control group: 24.8 h, with an IQR of 12 to 47 h) was also similar for both groups. Although all the neonates in the early iNO group received iNO, 81 of the 149 (54%) control subjects received standard iNO therapy.^10^ Receiver operating characteristic curve analysis determined that an OI of 20 was the optimum cut point for the prediction of ECMO/death risk by OI at the time of enrollment in both groups and was used to stratify infants in each group by severity of HRF (OI 15 to <20 vs OI 20 to 25).

### Univariate analysis for ECMO/death

Univariate analysis on the entire cohort identified that OI at enrollment <20 compared with ⩾20, primary diagnosis associated with HRF and surfactant therapy before randomization were significantly associated with a decreased risk of ECMO/death (Table 1). Univariate analysis of data by treatment groups revealed that for the infants in the early iNO group, an OI of <20 (10.2%) relative to OI of ⩾20 (25.8%, *P*=0.015) was associated with a decreased rate of ECMO/death. For the control group, exposure to surfactant therapy (13.4%) compared with lack of surfactant (30.8%, *P*=0.02) was associated with a decreased rate of ECMO/death.

### Multivariate logistic regression analysis for ECMO/death

Multivariate logistic regression analysis on the entire study cohort demonstrated that enrollment at an OI of <20, surfactant therapy and the diagnosis of RDS relative to primary pulmonary hypertension were associated with a decrease in the rate of ECMO/death (Table 2). PaO_2_ response and prior use of HFV were not significantly associated with the rate of ECMO/death. For infants randomized to the early iNO group, exposure to iNO at an OI of <20 was associated with a decreased rate of ECMO/death, compared with infants who received this therapy at an OI of ⩾20 (Table 2). For control infants, only surfactant use was associated with a decrease in the rate of ECMO/death.

### Effect of surfactant on the risk of ECMO/death by diagnosis in the entire study cohort

We analyzed the ECMO/death outcome by surfactant therapy for individual lung diseases associated with HRF, irrespective of exposure to iNO therapy. Surfactant use in infants with RDS, perinatal aspiration syndrome and pneumonia/sepsis was associated with a decrease in the rate of ECMO/death (Table 3). Surfactant did not alter the risk of ECMO/death for the infants with primary pulmonary hypertension. Surfactant use for the combined category of any lung disease other than primary pulmonary hypertension was associated with a threefold reduction in the risk of ECMO/death (Table 3). This interaction was highly significant by multivariate logistic regression analysis (*P*=0.003).

### Interaction of surfactant and iNO therapy for the risk of ECMO/death

Control infants treated with surfactant were less likely to progress to standard iNO therapy at OI ⩾25 (47 vs 67%, *P*=0.03) or to receive ECMO or die (13.4 vs 30.8%, *P*=0.02) than their non-treated counterparts. If surfactant-treated control infants progressed to standard iNO, their ECMO/death rate (26%) was lower than their non-treated counterparts (46%, *P*=0.10), although this difference was not statistically significant.

In the early iNO group, infants treated with surfactant experienced a similar rate of ECMO/death as their non-treated counterparts (13.7 vs 21.8%, *P*=0.21). The rate of progression to standard iNO at OI ⩾25 (36 vs 49%, *P*=0.12) and the rate of ECMO/death for infants who progressed to standard iNO therapy were also similar for infants who received surfactant and those who did not (35.3 vs 33.3%, *P*=1.0) in the early iNO group.

### Risk of ECMO/death by treatment assignment and OI at enrollment

Infants randomized to the early iNO group who received iNO at an OI of 15 to <20 (9/88, 10.2%) experienced a 60% relative reduction in ECMO/death compared with early iNO infants who received iNO at an OI of 20 to 25 (16/62, 25.8%, *P*=0.02). In contrast, control infants enrolled at the lower OI (16/92, 17.4%) had a 24% relative reduction in ECMO/death rate compared with those infants enrolled at OI of 20 to 25 (13/57, 22.8%, *P*=0.52), which was not significant. For infants enrolled at an OI of 15 to <20, the ECMO/death rate for those in the early iNO group (10.2%) was 41% lower than for the controls (17.4%); however, the difference was not statistically significant (*P*=0.16), nor was an interaction detected by logistic regression analysis (*P*=0.21).

### Progression of HRF

We analyzed this outcome as either progression to OI ⩾30 or a composite outcome of OI increase to ⩾30 and/or ECMO/death. For all infants treated with early iNO at an OI 15 to 25, progression of HRF to OI ⩾30 (early iNO, 16.7 vs control, 32.2%, p =0.002) or to the composite outcome of OI ⩾30 and/or ECMO/death (early iNO, 25% vs control, 38%, *P*=0.02) occurred less frequently compared with control infants.

### Other outcomes before discharge

**Figure 1:**
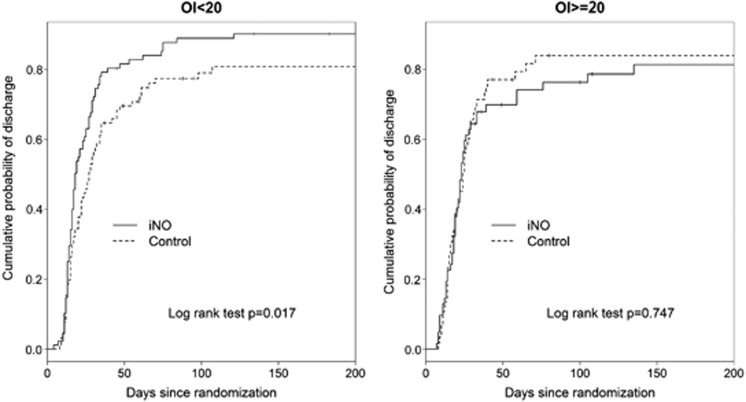
Kaplan–Meier survival analysis curves showing cumulative probability of discharge home for control and early inhaled nitric oxide (iNO) infants, grouped by oxygenation index (OI) at enrollment.

Among the entire cohort, administration of iNO for HRF at an OI of 15 to 25 did not alter the duration of mechanical ventilation, oxygen therapy and length of hospital stay as reported previously.^10^ However, early iNO group infants treated at a lower acuity of illness (OI 15 to <20) were discharged home sooner than control infants enrolled at the same OI (log rank test *P*=0.02, Figure 1). Thus, 50% of infants who received early iNO at OI <20 were discharged home by day 18, compared with day 27 for control infants enrolled at the same OI. Similarly, multivariate analysis using a Cox proportional hazards model indicated earlier discharge for early iNO infants treated at OI <20 compared with control infants enrolled at the same OI (Cox hazard ratio of 1.54 with CI 1.1 to 2.2) or early iNO infants treated at a higher OI of 20 to 25 (Cox hazard ratio 1.54 with CI 1.1 to 2.3).

Surfactant therapy was associated with a decreased number of days on mechanical ventilation (surfactant treated, median 8 days with IQR of 6 to 12 days vs no surfactant, median 9 days with IQR 7 to 15 days, *P*=0.05) and length of hospital stay (discharge home by 30 days, surfactant treated 68% vs no surfactant 58% and discharge home by 60 days, surfactant treated 82% vs no surfactant 68% *P*=0.04 by log rank test). The number of days on oxygen was similar between surfactant treated and non-treated infants.

## Discussion

In our previously reported RCT, introducing iNO therapy in moderate HRF (OI 15 to 25) had no effect on the ECMO/death rate or duration of hospital stay compared with the control group infants who received iNO therapy if their respiratory failure progressed to an OI of ⩾25.^10^ However, our study design provided little separation in the severity of illness at iNO initiation between the two study groups, as many infants in the early iNO group were very close to the threshold OI of ⩾25 for standard iNO at study enrollment. This *post-hoc* reanalysis of the subgroups stratified by OI at enrollment (OI 15 to <20 vs OI 20 to 25) identified several potential benefits of initiating iNO therapy at a lower acuity of illness among the original cohort. First, we observed that earlier initiation of iNO at OI <20 decreased the rate of ECMO/mortality and shortened the hospital stay, compared with later initiation at OI 20 to 25. These data suggest that initiation of iNO at an OI ⩾20 is relatively late in the course of HRF. These significant benefits were also suggested in some previous, smaller studies. The Franco–Belgian collaborative NO trial observed shortened duration of mechanical ventilation and time in the NICU for neonates treated earlier with iNO therapy, but did not detect a reduction in hospital stay.^18^ We also observed that the early iNO cohort who received this therapy at OI 15 to 25 experienced decreased progression of HRF to OI ⩾30 and to the composite outcome of OI ⩾30 and/or ECMO/death compared with standard application of this therapy at OI⩾25 in the control group. Progression to OI ⩾30 is an important parameter, as it provides time for transfer of neonates to an ECMO center or for consideration of ECMO before reaching standard ECMO criteria, based on OI >40.^19^ These data are similar to the report of González *et al.*^20^ who observed that early administration of iNO to term and late preterm neonates in HRF at an OI of 10 to 30 decreased their progression to OI >40. González *et al.*^20^ also observed that earlier application of iNO decreased the duration of oxygen therapy but did not report its effect on the duration of hospital stay. We did not observe a difference in duration of oxygen therapy with early administration of iNO, but found a decrease in length of hospital stay when it was given at OI <20. In addition, a pooled three-trial analysis by Golombek and Young^21^ reported a decrease in the duration of mechanical ventilation with iNO therapy, compared with a control group that did not receive this therapy (median duration of 11 days with iNO therapy vs 14 days in the control group) . Together, these studies suggest that earlier initiation of iNO therapy can decrease the progression of HRF and its complications. Both our study and that of González *et al.* (20) required infants to be on high FiO_2_ to be eligible for enrollment (FiO_2_ 0.80 and 1.0, respectively). Whether the initiation of iNO at a lower FiO_2_ can improve outcomes further requires additional study.

In our *post-hoc* analysis, early surfactant therapy for infants with parenchymal lung disease was associated with a striking, threefold reduction in the risk of ECMO/death in our study. Previously, Lotze *et al.*^11^ reported that surfactant therapy decreased the need for ECMO in term neonates born at ⩾36 weeks gestation with HRF in a RCT. This decrease was primarily observed in infants with meconium aspiration syndrome and sepsis, and not in infants with primary pulmonary hypertension. The beneficial effect of surfactant in their trial was also primarily seen for infants at an OI of 15 to <23, whereas infants with OI >30 experienced no benefit.^11^ Our data are consistent with the observations from the trial of Lotze *et al.*^11^ In addition, we found that the overall ECMO/death rate in our study for surfactant-treated babies (13.5%) was lower than the overall ECMO rate observed in surfactant-treated group (29.3%) in Lotze *et al.*'s^11^ trial, but similar to the subgroup treated at OI <23 in their trial. Taken together, these data suggest that surfactant is more effective when administered early in the course of HRF. Surfactant therapy was also previously shown to improve oxygenation, decrease the incidence of associated PPHN and shorten the duration of ventilation and hospital stay in term neonates with meconium aspiration syndrome.^12, 13, 14^ Our data from a large cohort of patients in the era of iNO therapy further reinforced these findings. In contrast, HFV did not confer such benefit in our study cohort.

The strengths of our present study are that it includes prospectively obtained data from a large study cohort of 299 term and late preterm neonates presenting with moderate HRF. This secondary analysis identified specific lung diseases associated with HRF that benefited from early surfactant therapy. As another randomized trial of surfactant is unlikely to be done in a large number of term and late preterm neonates with HRF, our observations provide important correlative data in support of its early use in HRF caused by parenchymal lung disease. Similarly, we identified a subgroup of neonates presenting at lower OI in HRF who appear to benefit from early use of iNO. These data can provide direction for a future RCT of iNO therapy early in the course of HRF.

The primary limitations of this data analysis are its retrospective nature and lack of randomization of surfactant use. It is possible that sicker babies were not given surfactant due to a concern of precipitating respiratory deterioration following surfactant. However, the mean OI at enrollment for the 192 babies who received surfactant (19.3±2.8) did not differ from the mean OI for the 107 babies who did not receive surfactant (19.6±2.8, *P*=0.36). The early iNO trial also did not specify the ventilator strategy and did not address whether the use of iNO with a specific mode of ventilation improved the outcome.

In conclusion, this *post-hoc* analysis of the early iNO RCT data revealed that administration of surfactant and iNO therapy at a lower acuity of illness was associated with a decreased risk of ECMO/death, progression of HRF and decreased duration of hospital stay. Whether iNO therapy is beneficial in less severe HRF, given through non-invasive respiratory support at lower FiO_2_ requires future investigation.

## Figures and Tables

**Table 1:** Univariate analysis for ECMO/death outcome for the entire study cohort consisting of early inhaled nitric oxide and control groups

*Variable*	*All infants (*n*=299)*	*No ECMO/death (*n*=245)*	*ECMO/death (*n*=54)*	P*-value*
Gestational age—mean (s.d.)	38.6 (2.0)	38.7 (2.0)	38.4 (2.0)	0.49
				
PaO_2_ response, torr				0.23
Median (IQR)	37.0 (4.0, 89.0)	38.0 (6.0, 89.0)	22.0 (-3.0, 92.0)	
Mean (s.d.)	61.5 (85.1)	62.7 (82.6)	56.5 (95.8)	
				
PaO_2_ response, n (%)				0.676
Unknown	5	5 (100)	0	
<10 torr	100	83 (83)	17 (17)	
10–20 torr	39	30 (77)	9 (23)	
>20 torr	155	127 (82)	28 (18)	
				
OI at enrollment, n (%)				0.031
<20	180	155 (86)	25 (14)	
⩾20	119	90 (76)	29 (24)	
				
Diagnosis, n (%)				0.014
Primary pulmonary hypertension	79	60 (76)	19 (24)	
RDS	52	49 (94)	3 (6)	
Perinatal aspiration	126	101 (80)	25 (20)	
Pneumonia/sepsis	41	35 (85)	6 (15)	
Lung hypoplasia	1	0	1 (100)	
				
Prior use of surfactant, n (%)				0.008
Yes	192	166 (86.5)	26 (13.5)	
No	107	79 (74)	28 (26)	
				
Prior use of HFV, n (%)				0.88
Yes	130	106 (81.5)	24 (18.5)	
No	169	139 (82)	30 (18)	

Abbreviations: ECMO, extracorporeal membrane oxygenation; HFV, high-frequency ventilation; IQR, interquartile range; OI, oxygenation index; RDS, respiratory distress syndrome.

The *n* (%) for variables 3 to 7 in the table refer to the number and percent of neonates meeting the outcome specified for that column for each variable.

**Table 2:** Multivariate logistic regression analysis for ECMO/death outcome for the entire study cohort and for the individual treatment groups

*Variable*	*Odds ratio (95% CI) for ECMO/death*	P*-value*
*Entire cohort*		
Oxygenation index <20	0.52 (0.27–0.97)	0.04
Surfactant use	0.47 (0.24–0.91)	0.03
		
*Lung diseases relative to primary pulmonary hypertension*		
RDS	0.22 (0.05–0.75)	0.03
Perinatal aspiration	0.84 (0.41–1.75)	0.63
Pneumonia/sepsis	0.49 (0.16–1.37)	0.20
		
*Control group*		
Surfactant use	0.27 (0.10–0.72)	0.01
		
*Early iNO group*		
iNO at OI <20 relative to iNO at OI 20–25	0.25 (0.08–0.67)	0.01

Abbreviations: CI, confidence interval; ECMO, extracorporeal membrane oxygenation; iNO, inhaled nitric oxide; OI, oxygenation index; RDS, respiratory distress syndrome.

**Table 3:** Effect of surfactant on the risk of ECMO/death for lung diseases associated with HRF among the entire study cohort consisting of both early inhaled nitric oxide and control groups

Diagnosis	*ECMO/death for surfactant-treated group,* n *(%)*	*ECMO/death for the no surfactant group,* n *(%)*	P*-value*
Primary pulmonary hypertension	12/42 (28.6)	7/37 (19)	0.43
RDS	1/45 (2.2)	2/7 (28.6)	0.04
Perinatal aspiration syndrome	11/79 (14)	14/47 (30)	0.04
Pneumonia/sepsis	1/25 (4)	5/16 (31)	0.03
Lung diseases other than primary pulmonary hypertension	14/150 (9.3)	21/70 (30)	<0.001

Abbreviations: ECMO, extracorporeal membrane oxygenation; HRF, hypoxic respiratory failure; RDS, respiratory distress syndrome.

Data are shown for babies who met ECMO/death outcome for each diagnosis, for surfactant-treated and untreated groups as number (*n*) and percent (%).

## Notes

Dr G Ganesh Konduri received equipment for inhaled nitric oxide for his research in pulmonary hypertension, was site PI for a clinical trial of iNO supported by Ikaria and received an unrestricted grant from Ikaria to his institution to support the secondary analysis of data presented in this manuscript. Dr Greg Sokol was the site PI for a clinical trial supported by Ikaria. Dr Krisa Van Meurs was the site PI for a clinical trial supported by Ikaria. Dr N Ambalavanan received a research grant from Ikaria in 2010. The remaining authors declare no conflict of interests.
